# Improving the uptake of contraception, Somalia 

**DOI:** 10.2471/BLT.23.290299

**Published:** 2023-12-08

**Authors:** Md Nuruzzaman Khan, Ibrahim Yasin Khalif, Md Shohel Rana, Md Mostaured Ali Khan, Shimlin Jahan Khanam, Md Badsha Alam

**Affiliations:** a Department of Population Science, Jatiya Kabi Kazi Nazrul Islam University, Mymensingh-2220, Bangladesh.; b Faculty of Economics, Somali National University, Mogadishu, Somalia.; c Maternal and Child Health Division, icddr,b, Dhaka, Bangladesh.

The 2020 Somali Health and Demographic Survey showed that maternal mortality as well as that of children younger than five years, is very high in the country, with 692 deaths per 10 0000 population and 71 deaths per 1000 population, respectively.^1^ Furthermore, malnutrition affects one fourth of the Somali population.^1^ The total fertility rate in Somalia remains elevated at 6.9 children per woman, compared to 4.15 in Africa and 2.30 worldwide. The health survey estimates that unintended and short-interval pregnancies are also common. These unfavourable indicators place Somalia among the worst countries globally in terms of sexual and reproductive health indicators, as well as in maternal and child health.

These figures also reveal that despite the successful implementation of the millennium development goals between 2000 and 2015^2^ and the sustainable development goals being halfway to the 2030 target year, very little improvement is observed in Somalia. The country is significantly lagging behind in achieving these goals.

## What needs to be done?

Addressing these critical challenges requires prioritizing the availability, accessibility and use of modern contraception. However, Somalia falls considerably short of attaining these objectives. The recently published findings of the 2020 Somali Health and Demographic Survey^1^ reveal a concerning situation. Only an estimated 7% of women of reproductive age use any form of contraception, and less than 1% use modern contraceptive methods such as pills, condoms and intrauterine devices.^1^ Additionally, unmet need for contraception is reported at 37%.^2^ Therefore, Somalia has one of the lowest rates of contraception uptake in the world. Significant determinants of contraception use and unmet need for contraception include age; education level; number of children; exposure to family planning messages through mass media; and region and location of residence. Given the connection between contraception and other maternal and child health outcomes, including maternal and child mortality, these findings indicate that Somalia is unlikely to see improvements in maternal and child health, including maternal and child mortality, in the coming years unless initiatives to increase uptake of modern contraception are taken.

## Challenges

The prolonged political instability, armed conflicts and widespread poverty in Somalia has resulted in an underdeveloped health-care infrastructure and a severe shortage of health-care personnel.^3^ Discussions about family planning and providing contraception often require privacy and need to be conducted in separate areas. However, dedicating private areas for this purpose is nearly impossible with the existing poor health-care systems, which primarily prioritize emergency health-care services, including the treatment of serious maternal health complications. The prevailing social norms surrounding family planning, contraception and the desired number of births, along with misconceptions about contraceptive use such as viewing contraception as a sin, pose formidable constraints. Deeply ingrained societal and cultural norms that perceive having additional children as a blessing prevail.^4^ Women in Somalia also fear that failing to bear children as per their partner’s wishes could lead to losing their partner – a fear mainly motivated by longstanding illiteracy and the tradition of polygamy.^4^ The limited access to education severely affects women’s knowledge and awareness regarding the importance of contraception use, and their involvement in reproductive decision-making processes.^5^ Access to alternative sources of family planning and contraception information, such as mass media including radio, television and newspapers, is severely limited in Somalia.^1^ Community and religious leaders in Somalia also hold several misconceptions about fertility, birth spacing and family planning.^6^ Moreover, evidence exists of significant misconceptions among health-care providers, who discourage women from using contraception unless they have specific health concerns.^7^ This situation diverges from other countries where health-care providers play a pivotal role in advocating for and facilitating increased contraceptive uptake.^8^

## Solutions

To address these issues, Somalia needs to prioritize sexual and reproductive health-care services, with a particular emphasis on improving access to modern contraception.^9^ Achieving this goal requires a multifaceted approach, including the implementation of comprehensive education programmes to raise awareness about contraception, and challenge prevailing social and cultural norms regarding family planning.^10^ Mobilizing the community and fostering greater engagement are vital components of increasing awareness, and promoting the importance of family planning and contraception.^11^ Additionally, the development of multisectoral programmes involving various stakeholders is crucial for ensuring widespread access to contraception. However, acknowledging that this endeavour may not be immediately feasible for the Somali government is important. The primary reason for these challenges lies within the health-care system in Somalia, currently ranked as one of the weakest in the world, with only one third of the population having access to essential health-care services.^1^ This situation is further exacerbated by inadequate government funding; relying solely on international donors cannot bridge this gap.^1^

One solution would be to implement Bangladesh’s family planning model in Somalia. In 1971, following the country’s independence, Bangladesh had a contraception uptake of only 8% (out of the 4 million women of reproductive age).^10^ Lack of access to contraception and limited awareness about contraception methods were the major challenges. After 9 months of prolonged war following years of deprivation, the health-care infrastructure was inadequate. Moreover, Bangladesh faced numerous social and religious misconceptions regarding contraception uptake, being a country with a majority of Muslim population.^9^ These demographic and historical circumstances bear similarities to the current situation in Somalia. However, Bangladesh developed a unique approach to providing family planning services that helped reduce the country’s fertility rate to replacement level by 2022, with contraception uptake reaching around 62% in the span of 40 years.^11^ The underlying concept of this model was to establish a separate directorate under the health ministry dedicated solely to family planning and contraception. The directorate recruited secondary-educated women, provided them with short training, and tasked them with visiting every couple’s home within a small geographical area in a 14-day cycle.^9^

By adopting a similar approach, the Somali government could address family planning issues at the grassroots level by using women with a general educational background, allowing expert health-care personnel to focus on providing other essential health-care services. Moreover, this approach would alleviate pressure on health-care infrastructure and ensure the efficient delivery of contraception services. Conducting home visits to discuss family planning, contraception, misconceptions and taboos would enable addressing the major challenges that Somalia currently faces in women’s unmet need for contraception as well as in the health-care system. 
